# One-dimensional flat bands in twisted bilayer germanium selenide

**DOI:** 10.1038/s41467-020-14947-0

**Published:** 2020-02-28

**Authors:** D. M. Kennes, L. Xian, M. Claassen, A. Rubio

**Affiliations:** 1 Institut für Theorie der Statistischen Physik, RWTH Aachen University and JARA-Fundamentals of Future Information Technology, 52056 Aachen, Germany; 2 Center for Free Electron Laser Science, Max Planck Institute for the Structure and Dynamics of Matter, 22761 Hamburg, Germany; 3 Center for Computational Quantum Physics, Simons Foundation Flatiron Institute, New York, NY 10010 USA; 4 Nano-Bio Spectroscopy Group, Departamento de Fisica de Materiales, Universidad del País Vasco, UPV/EHU-20018, San Sebastián, Spain

**Keywords:** Electronic properties and materials, Two-dimensional materials, Magnetic properties and materials, Phase transitions and critical phenomena

## Abstract

Experimental advances in the fabrication and characterization of few-layer materials stacked at a relative twist of small angle have recently shown the emergence of flat energy bands. As a consequence electron interactions become relevant, providing inroads into the physics of strongly correlated two-dimensional systems. Here, we demonstrate by combining large scale ab initio simulations with numerically exact strong correlation approaches that an effective one-dimensional system emerges upon stacking two twisted sheets of GeSe, in marked contrast to all moiré systems studied so far. This not only allows to study the necessarily collective nature of excitations in one dimension, but can also serve as a promising platform to scrutinize the crossover from two to one dimension in a controlled setup by varying the twist angle, which provides an intriguing benchmark with respect to theory. We thus establish twisted bilayer GeSe as an intriguing inroad into the strongly correlated physics of lowdimensional systems.

## Abstract (web summary)

Twisting the relative orientation of the sheets in few-layer van der Waals materials can cause drastic changes in the electronic bandstructure. Here, the authors predict that twisted bilayer GeSe realises an effective one-dimensional flat-band electronic system with exotic, strongly correlated behaviour.

## Introduction

Understanding emergent, strongly correlated quantum phenomena in complex many-body interacting and low dimensional materials is one of the main driving forces in modern condensed matter research. Strongly correlated systems are fascinating as they challenge our understanding of quantum mechanics fundamentally, but are also highly relevant to technological advances, such as the quest for room temperature superconductivity, ultra-dense and ultra-fast memory solutions, as well as quantum computing platforms^1^, to name a few. In this context, the study of low-dimensional systems has revealed a zoo of surprising insights into quantum collective behavior of many-body systems, some of which already find far reaching applications in everyday life, e.g., in computer memory (magnetism) and magnetic resonance imaging techniques (superconductivity).

Recently, twisted bilayer graphene^2–6^ and other van der Waals materials stacked atop each other at a twist^7–13^ have been proposed as material realizations of two-dimensional correlated physics that afford an unprecedented level of control. Previous studies concentrate on few-layer films featuring a $6{0}^{\circ }$ or $12{0}^{\circ }$ rotational symmetry stacked at a twist. By forming a large moiré supercell at small twist angles, a quasi-two-dimensional system with quenched and tunable kinetic energy scales emerges, thereby drastically enhancing the role of electronic interactions.

Surprisingly, we report here that if instead we consider layered systems stacked at a small twist angle for which the monolayers have a rectangular lattice with only mirror symmetry, an effectively one-dimensional system with quenched kinetic energy scales (flat bands) emerges. This elevates the concept of moiré systems to include the broad and exciting realm of one-dimensional quantum systems, which from a theory point of view is ideal to study quantum many-body effects, because powerful theoretical tools (such as bosonization, tensor network approaches and the Bethe ansatz^14–16^) can be employed to obtain a nearly complete picture of its collective nature and effects of strong correlations. Remarkably, we find that varying the twist angle smoothly interpolates between an effectively one-dimensional and a two-dimensional system at low energies, permitting experimental studies of the dimensional crossover in a clean and controllable manner.

**Fig. 1:**
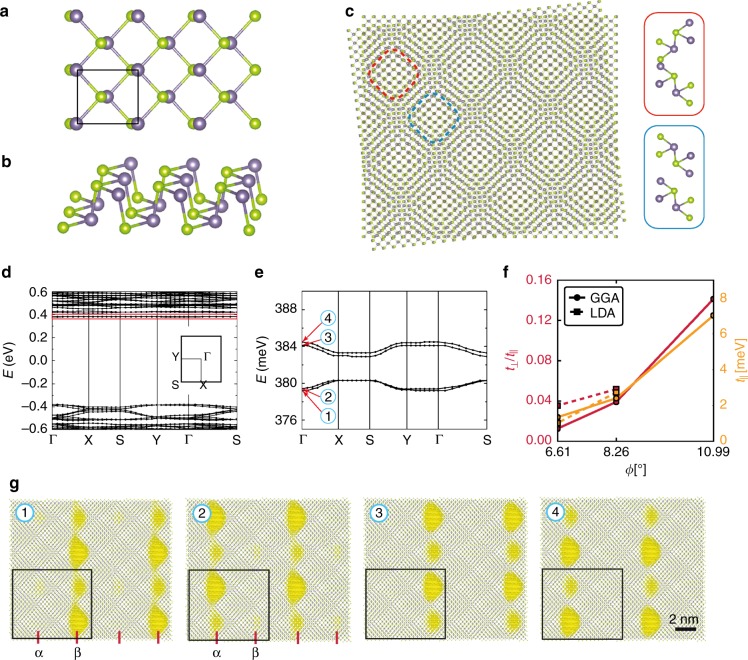
Ab initio characterization of twisted bilayer GeSe. **a**, **b** Top and side view of monolayer GeSe. Green and blue spheres indicate Se and Ge atoms, respectively. The black box in the top panel denotes the rectangular unit cell of the system. **c** moiré pattern for two sheets of GeSe stacked at a relative twist of 180-6.61 degree denoted by configuration B. The pattern that emerges shows a rectangular shape, with much larger unit cell. We highlight two areas with dashed lines whose staking is given in the right panels. **d**, **e** Band structure as obtained from density functional theory using the LDA. Flat bands emerge at the edge of the valence and the conduction bands, where **e** shows a zoom into the red-boxed region highlighted in **d**. The flat bands at the conduction band disperse only along one spatial direction, the $\Gamma \to X$ and $S\to Y$ direction. **f** LDA and GGA results for the ratio between inter-wire (${t}_{\perp }$) and intra-wire (${t}_{\parallel }$) couplings of the emergent one-dimensional chains at low energies as a function of twist angle, highlighting the emergence of quasi-one-dimensional physics at small twists. **g** Real space illustration of the one-dimensionality of the system showing the charge density of the bands labeled by 1–4 in **e** as accumulated yellow regions (the unit cell hosts a pair of wires with a staggered chemical potential and a wire-wire coupling that vanishes as the angle is decreased). The charge density wires are highlighted with red lines and annotated by $\alpha$ and $\beta$.

To illustrate this point we perform large-scale ab initio based simulations of two sheets of GeSe stacked at a twist, where GeSe belongs to the family of 2D group-IV monochalcogenides^17^ and has a similar structure as phosphorene [see Fig. 1a, b]. 2D GeSe exhibits high air stability and thin GeSe films down to a monolayer have been studied extensively in experiments for their applications in phototransitors and near-infrared photodetectors^18–24^. 2D GeSe is also predicted to exhibit giant piezoelectricity^25,26^, room-temperature ferroelectricity^27,28^ and ferroelasticity^29,30^, strong visible-light absorbance^31^ and a large bulk photovoltaic effect^32^. This renders GeSe an interesting choice as much prior expertize on the (untwisted) material exist and samples are experimentally available. Furthermore, recently an Eshelby twist has already been realized for GeSe, as well as in the structurally similar system GeS^33^.

With extensive ab initio calculations, we explicitly demonstrate that a quasi-one-dimensional system emerges for twisted bilayer GeSe at small twist angles, where the degree of “one-dimensionality” increases with decreasing angle. Upon including interactions we show that this system is an effective realization of the so-called ionic Hubbard model. This model has attracted a lot of research attention in the past^34–40^, because it features many interesting prototypical (correlated) phases of matter, including band insulators, Mott insulators, bond density waves and Luttinger liquids, and hosts Ising, as well as Kosterlitz–Thouless quantum phase transitions. As a consequence we find that in twisted GeSe all these different phases of matter can be accessed and their respective phase transitions can be studied in a controllable condensed matter setup. We explicitly outline the phase diagram including all the above mentioned phases of matter upon varying the filling (experimentally tunable by gating), as well as the ratio of kinetic and interaction energy scales (tunable by the twist angle) at temperatures accessible within current experimental limitations. Furthermore, twisted bilayer GeSe constitutes a unique system for the controlled study of the crossover between two-dimensional and one-dimensional physics via varying the twist angle using the experimental setup outlined in ref. ^41^, which can be used to shed light on this interesting regime from an experimental viewpoint in the future. This condensed matter based benchmark system could complement results from more conventional quantum simulation platforms^42–44^ in the future in terms of scalability of system size and operation temperature. Twisted bilayer GeSe, as we demonstrate, is thus an ideal inroad into the strongly correlated nature of low-dimensional systems.

## Results

### Density functional characterization

We start by discussing the ab initio band structure results for twisted GeSe. In Fig. 1 we show the density functional theory (DFT) characterization of two sheets of stacked GeSe at a twist (see Methods section). The atomic structure of a single sheet of GeSe resides in a rectangular lattice [panels (a) and (b)]. Starting from a perfectly aligned AA-stacking bilayer, different moiré patterns are formed when the top (or the bottom) layer is twisted with angle $\phi$ ranging from ${0}^{\circ }$ to $18{0}^{\circ }$ with respect to the other layer.

The systems with twist angles $\phi$ and $18{0}^{\circ }$-$\phi$, which we refer to as configurations A and B, respectively, share supercells of the same size. The supercell for system B is shown in Fig. 1c, and the corresponding supercell for system A can be found in the method section. We will focus on configuration B in this work and we refer to the twist angle of $18{0}^{\circ }$-$\phi$ as $\phi$ for simplicity. Similar to the results reported for hexagonal or triangular lattice systems^2–6,8,9,45^ we find the emergence of flat bands (which as in the case of twisted Boron-Nitride^8^ does not rely on tuning to magic angles) at the edges of the conduction and valence bands at small twist angles. However, in marked contrast to these other systems surprisingly some of the low energy bands disperse only along one direction in real space. This is most obvious for bands at the bottom of the conduction bands [see panel (d) and (e)], which are only dispersive along the $\Gamma$-X (or Y-S) direction and dispersionless along the perpendicular $\Gamma$-Y (or X-S) direction. We carefully checked these results against varying the functionals used in our DFT calculations, which give slightly different relaxed atomic geometry. To this end we compare results obtained within the local density approximation (LDA) to those obtained employing a generalized gradient approximation (GGA) with van der Waals corrections. We find very consistent behavior upon varying the choice of functionals (see Methods section). Remarkably, we find that the moiré system at small angle shows a quasi-one-dimensional chain-like staggered charge distribution in real space [see panel (g)] for states in the flat bands, with pairs of wires in the unit cell, each of which displays an alternating sequence of large and small charge puddles. To capture this behavior, we fit the low-energy moiré bands using an anisotropic tight-binding model with a staggered on-site potential (see Methods section). Panel (f) summarizes results for such fits obtained within a LDA and GGA. We find (robust to changing the functionals used in DFT) that the ratio between intra-wire and inter-wire couplings decreases with decreasing twist angle, which tunes the system continuously to the one-dimensional limit.

If we neglect the coupling between the one-dimensional wires at small twist angle, then a simple model that accurately describes the dispersion and charge modulation along the wire is given by a Hamiltonian with nearest-neighbor hopping $t$ and featuring a staggered on-site potential ${\epsilon }_{0}$

1 $${H}_{0,\sigma }=\sum _{i}t\ {c}_{i,\sigma }^{\dagger }{c}_{i+1,\sigma }+{\rm{H}}.{\rm{c}}.+\sum _{i}{(-1)}^{i}{\epsilon }_{0}{n}_{i,\sigma },$$

with ${n}_{i,\sigma }={c}_{i,\sigma }^{\dagger }{c}_{i,\sigma }$ the occupancy at site $i$. The corresponding dispersion has two branches ${E}_{k}^{\pm }={\pm} \!\sqrt{4{t}^{2}{\cos }^{2}(k)+{\epsilon }_{0}^{2}}$.

**Fig. 2:**
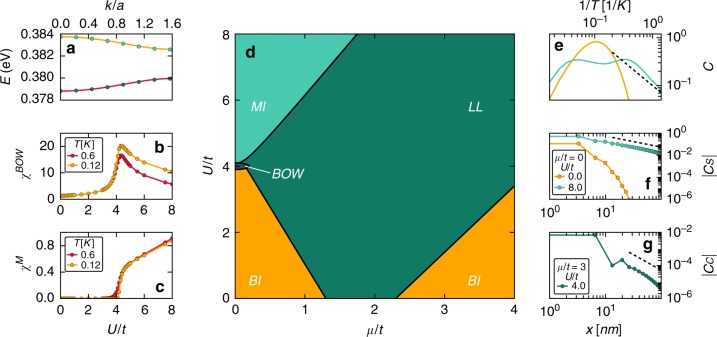
Characterization of many-body electron correlations in twisted bilayer GeSe. **a** Fit (solid lines) to the ab initio results shown in Fig. 1. The fit yields parameters $t=1.03$ meV and ${\epsilon }_{0}/t=1.3$ for $\phi =6.6{1}^{\circ }$. **b**, **c** Susceptibilities for bond order, as well as magnetization are used to map out the phase boundaries between the Band insulator (BI), the bond ordered wave (BOW) state and the Mott insulating (MI) state at half filling $\mu =0$. The first transition (BI $\to$ BOW) is a continuous Ising phase transition, while the second (BOW $\to$ MI) is of the Kosterlitz-Thouless type^34–38^. Upon doping the system away from half filling the system turns to a gapless Luttinger liquid state (at non-zero $U$) characterized by critical power-law correlations in spin and charge degrees of freedom. The full phase diagram at $T=0$ is summarized in **d**. **e** Specific heat and (**f**) spin-spin correlation function at half filling for two values of $U$, placing the system either in the band insulating or Mott insulating state, respectively. The specific heat (**e**) at large inverse temperatures $1/T$ turns from exponential (BI) to linear (MI) which is a hallmark of gapless spin excitations in the MI state. The double maxima structure in $c$ is a hallmark of the lower and upper Hubbard band^57^. We find that at $1K$ the system starts to show clear MI behavior (specific heat $c$ turns linear) for $U/t=8$. Panel **f** shows the spin-spin correlation function. In the BI phase we find exponential suppression, while in the MI state the state shows long range algebraic correlations ${C}_{S} \sim {x}^{-1}$ at $T=1/8K$. Panel **g** shows the charge-charge correlation function obtained for finite doping $\mu /t=3$. The long-ranged power-law decay (dashed line) in the correlation functions falls of as approximately ${C}_{S} \sim {x}^{-1.9}$ which is indicative of a weakly correlated Luttinger liquid (Luttinger parameter ${K}_{C}=0.95$) at this $U/t=4$.

Starting from the LDA DFT results at a twist angle of $\phi =6.6{1}^{\circ }$, ${\epsilon }_{0}=0.001337$eV can be read off by the gap magnitude at the zone edge, and an optimal fit of the single remaining parameter $t={t}_{\parallel }$ (see Methods section) is shown in Fig. 2a. At this angle we find ${\epsilon }_{0}/t\approx 1.3$, placing the system in the interesting regime where kinetic energy terms and staggering potential compete in their order of magnitude. More details about the fit, as well as the crossover from two dimensions to one can be found in the Methods section.

### Correlation effects

Next, we model electron interaction effects. At this point we have no definite way to pinpoint the range of the interactions and rather adopt the vantage point that screening will promote rather short ranged interactions. To this end we include an on-site repulsion with

2 $${H}_{U}=U\sum _{i}({n}_{i,\uparrow }-1/2)({n}_{i,\downarrow }-1/2),$$

as the dominant contribution. The interactions are written in a particle-hole symmetric way for convenience which amounts to an overall shift in chemical potential. This model is known in the literature as the ionic Hubbard model; a paradigmatic model to study the transition from band insulators (BI) to Mott insulators (MI) as the interactions are increased and was investigated extensively at half-filling^34–40^. It is now well understood that this transition occurs via an intermediate bond order wave state (BOW), in which interaction induced spontaneous dimerization leads to alternating strong and weak bonds. The transition from BI to BOW is of the Ising, second order type, while the second transition from the BOW to the MI state is of the Kosterlitz-Thouless (KT) type^34–38^. Twisted GeSe thus provides an inroad into this highly intriguing physics and can, depending on the parameters, potentially realize all of these different phases. So far we have used a (zero temperature) ab initio analysis of the band structure. For experiments, however, an important question is whether and how the emergent, correlated phases manifest at finite but still low temperature. This can be simulated efficiently for any chemical potential $\mu$, as well as $U$ and ${\epsilon }_{0}$ using density matrix renormalization group (DMRG) (see Methods section) taking the ab initio band structure as an input (at higher temperature the band structure itself might be affected but this regime is not the one we focus on in this work).

Much is known about the phases in the ionic Hubbard model and how to characterize them^34–38^, particularly at half-filling. We summarize how to distinguish these phases in Table 1, where we characterize the four different phases band insulator, bond ordered wave, Mott insulator and Luttinger liquid by whether they display a charge gap, spin gap and a staggered bond dimer order. A checkmark signals that the phase displays a non-zero value of the gap or order, while a cross denotes the absence thereof. By calculating the static susceptibility to magnetization ${\chi }^{M}$, charging ${\chi }^{C}$ and bond ordering ${\chi }^{BOW}$ upon including a small seed perturbation in magnetic field, onsite potential or bond dimerization, respectively, we determine the spin and charge gaps, as well as the bond ordering tendencies (see Supplementary Note 1). For the smaller angle of $\phi =6.6{1}^{\circ }$, we show ${\chi }^{M}$ and ${\chi }^{BOW}$ given a small seed $s/t=1{0}^{-2}$ in Fig. 2b, c. By calculating the static susceptibilities in this fashion and varying $U/t$, as well as $\mu /t$ (corresponding experimentally to controlling the angle, as well as back gate) we can map out the phase diagram by using Table 1. Panel (d) of Fig. 2 shows the full phase diagram we obtain this way. The BOW state occupies only a tiny fraction of the phase diagram and most likely requires fine tuning to be seen in experiments, especially at finite temperature.

**Table 1:** Theoretical characterization of the different phases of matter that can be realized in twisted GeSe^34–38^.

	Band insulator	Bond ordered wave	Mott insulator	Luttinger liquid
Charge gap	✔	✔	✔	✘
Spin gap	✔	✔	✘	✘
Bond dimer	✘	✔	✘	✘

The four different phases band insulator, bond ordered wave, Mott insulator and Luttinger liquid are distinguished by whether they display a charge gap, spin gap and a staggered bond dimer order. A checkmark signals that the phase displays a non-zero value of the gap or order, while a cross denotes the absence thereof.

The different phases of matter manifest prominently in transport experiments with the insulating gap scaling either with ${\epsilon }_{0}$ or $U$ in the BI and MI case, respectively, while showing characteristic power-law suppression in temperature in the LL regime. Scanning tunneling microscopy (STM) will reveal either a charge gap (BI and MI) with different temperature scaling or a power-law suppression of the density of states in the LL case. Both transport and STM have recently been successfully put forward in the twisted van der Waals material’s context^2–6^. Furthermore, specific heat and spin-spin correlation functions can be monitored to distinguish between these phases. In panel (e) and (f) of Fig. 2 we show the specific heat $c=\partial E/\partial T$, as well as the spin-spin correlation ${C}_{S}(x)$ at half filling for two values of $U/t=0$ (BI) and $U/t=8$ (MI). The specific heat in (e) at large inverse temperature $1/T$ is exponentially suppressed in the BI case while for a MI we find a linear behavior which is one of the hallmarks of the emergent gapless spin-excitations. We find that at $1K$ the system starts to show clear MI behavior (specific heat $c$ turns linear) for $U/t=8$. Panel (f) depicts the real space spin-spin correlation function. The BI phase is characterized by an exponential suppression of these correlation functions, while one of the hallmarks of the MI state are long range algebraic correlations ${C}_{S} \sim {x}^{-1}$ at $T=1/8K$, at least for small enough distances compared to $1/T$ (after which correlations fall off exponentially). We complement this by studying the charge-charge correlation function ${C}_{C}$ obtained for finite doping $\mu /t=3$ shown in panel (g). The long-ranged power-law decay (dashed line) in the correlation functions falls of as approximately ${C}_{S} \sim {x}^{-1.9}$ which indicates a weakly correlated LL state. Importantly, the temperatures for which all of these predictions can be measured are on the Kelvin scale and thus within experimental reach.

**Fig. 3:**
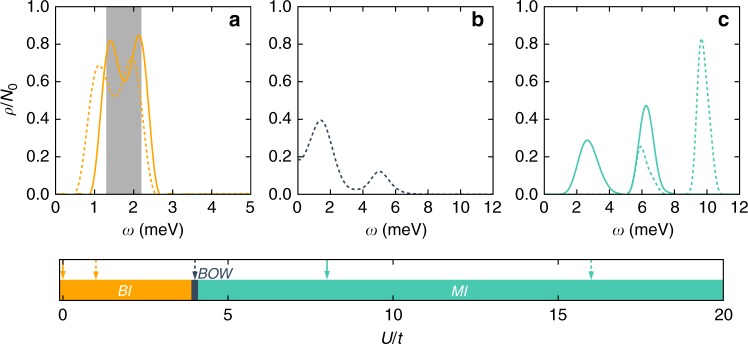
Density of states in twisted bilayer GeSe at $\mu =0$ obtained from DMRG. The bottom scale shows the different phases found in dependency of $U/t$ at half filling $\mu =0$. Arrows indicated the vales $U/t$ used to calculate the density of states shown in the upper panels ($U/t=0,1$ in **a**, $U/t=4$ in **b** and $U/t=8,16$ in **c**), which are grouped corresponding to the phases (BI, BOW or MI in **a**, **b** or **c**, respectively). In **a** a shaded region indicates the position of the non-interacting band edges, which agrees well with our numerics, where the density of states is found via real-time propagation. Consistent with Fig. 2, we find a non-monotonic gap size in the density of states as $U/t$ is increases, first decreasing and then increasing. Close to $U=0$ the gap is determined by ${\sim} {\epsilon }_{0}$ while at large $U$ it scales ${\sim} U$. The temperature in these calculations are $T=1.2K$ for **a**, **c**, as well as $T=2.4K$ for **b**. (Here ${N}_{0}$ normalizes the integral over the density of states to one).

Next, we highlight the signatures accessible via STM. We compute the density of states $\rho$ at the even lattice sites $i$ by simulating the real time dynamics of $\langle {c}_{i,\uparrow }{c}_{i,\uparrow }^{\dagger }(t)\rangle$ and taking the Fourier transform. Via the dissipation fluctuation theorem the local density of states can be obtained from this by dividing out the Fermi-distribution $f(-\omega )$ (see Methods for details). The results are summarized in Fig. 3 for temperatures in the Kelvin regime. At small $U$ we find that the single particle gap scales with ${\sim} {\epsilon }_{0}$, while the Mott insulating gap scales as ${\sim} U$. Overall the behavior of the gap first decreases (with a minimum close to the BOW phase) and then increases as $U$ is increased. The spectral features of the density of states can be used to clearly distinguish experimentally which phases are realized in the system.

## Discussion

We have established that twisted bilayer GeSe is an exciting platform to study strongly correlated one-dimensional physics and the crossover from one to two dimensions in a highly tunable manner. We find that upon marrying ab initio materials characterization and strong correlations a one-dimensional ionic Hubbard Model arises, which shows many prototypical features and phases of strongly correlated one-dimensional systems. These can be probed by experiments on twisted bilayer GeSe in accessible temperature regime, albeit on much enlarged moiré length scales. In twisted bilayer GeSe at small twist angles the spin-orbit splitting for the effectively one-dimensional system is negligible. Future research should address the questions whether in other moiré systems a stronger spin-orbit coupling can be realized. If so this would provide a highly controllable platform to realize Majorana edge state in these effective wires, by coupling the system to a conventional $s$-wave superconducting substrate.

In the final stages of writing this article ref. ^46^ appeared, which supports the message of this paper.

## Methods

### Details about the DFT treatment

**Fig. 4:**
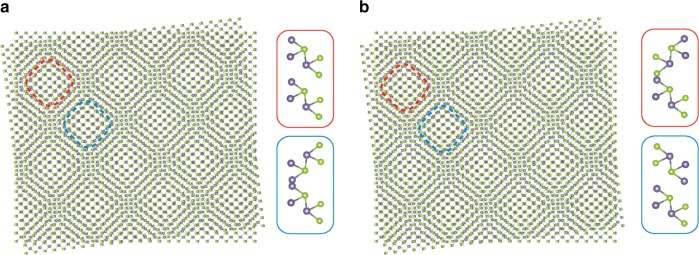
The two configurations of twisted bilayer GeSe in real space: **a** configuration A and **b** configuration B. They are related by a $18{0}^{\circ }$ rotation of the top layer and share the same size of supercell. The insets show the local atomic arrangements in the regions highlighted in red and blue in the main figures.

We employed the Vienna Ab initio simulation package (VASP) to perform the ground state DFT calculations^47^. The basis was chosen to be plane waves with an energy cutoff of 450 eV and the pseudo potentials are generated using the projector augmented wave method (PAW)^48^. The exchange-correlation functions are treated in the local density approximation (LDA)^49^. We complement our calculations by also considering the exchange-correlation functionals treated in the generalized gradient approximation (GGA)^50^ and find results consistent with LDA. A 1 × 1 × 1 momentum grid is used for the ground state and relaxation calculations. The experimental lattice constants for bulk GeSe (*a* = 4.38 ${\AA}$, *b* = 3.82 ${\AA}$) are employed for the construction of the supercell of twisted bilayer GeSe. In order to satisfy the commensurate condition, the a lattice constant is slightly expanded by 0.68%. As periodic boundary condition are applied, a vacuum region larger than 15 ${\AA}$ is added in the z-direction perpendicular to the layers to avoid artificial interaction between the periodic slabs. We relax all the atoms in order to avoid artificial effects as known from unrelaxed structures for other moiré systems^51–53^. Throughout the relaxation, all the atoms are relaxed until the force on each atom converges to values smaller than 0.01 eV/${\AA}$. In the GGA calculations, van der Waals corrections are applied using the DFT-D3 method of Grimme^54^. To visualize the charge density distributions of the low-energy states of twisted bilayer GeSe we employ the VESTA code^55^. There exist two inequivalent configurations called A and B in the main text, which are illustrated and characterized in Fig. 4. The supercell of twisted bilayer GeSe with twist angles at 10.99${}^{\circ }$, 8.26${}^{\circ }$ and 6.61${}^{\circ }$ contain 872, 1544 and 2408 atoms, respectively.

### Details about the fitted band structure and 1D-2D crossover

We use a simple tight binding model to describe the dispersion at all angles calculated within DFT. We consider a next-nearest-neighbor lattice model on a rectangular grid with a 2 by 2 sites unit cell:

3 $${H}_{0}=	 \sum _{i}{t}_{\parallel }\ {c}_{{i}_{x},{i}_{y}}^{\dagger }{c}_{{i}_{x}+1,{i}_{y}}+{\rm{H}}.{\rm{c}}.\\ 	 +{t}_{\perp }\ {c}_{{i}_{x},{i}_{y}}^{\dagger }{c}_{{i}_{x},{i}_{y}+1} +{\rm{H}}.{\rm{c}}.\\ 	 +{t}_{D}{c}_{{i}_{x},{i}_{y}}^{\dagger }{c}_{{i}_{x}+1,{i}_{y}+1}+{t}_{D}{c}_{{i}_{x},{i}_{y}}^{\dagger }{c}_{{i}_{x}-1,{i}_{y}+1}+{\rm{H}}.{\rm{c}}.\\ 	 +{\epsilon }_{i}{n}_{i},$$

with ${n}_{i,}={c}_{i}^{\dagger }{c}_{i}$ the occupancy at site $i=({i}_{x},{i}_{y})$. We fit the dispersion varying the nearest-neighbor hopping amplitudes along the $x$ direction (${t}_{\perp }$), along the $y$ direction (${t}_{\parallel }$), the next-nearest hopping along the diagonal (${t}_{D}$), as well as the onsite potentials ${\epsilon }_{i}$. We consider a 2 by 2 unit cell so ${\epsilon }_{i}$ can take 4 different values $({\epsilon }_{0,0},{\epsilon }_{1,0},{\epsilon }_{0,1},{\epsilon }_{1,1})$

**Fig. 5:**
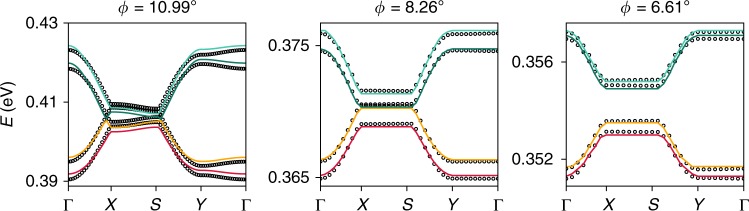
Ab initio band structure obtained with the GGA functionals and including van der Waals corrections (lines), as well as a next-nearest neighbor Hubbard model fit (circles) to the dispersion for different angles. This allows to extract nearest-neighbor hopping amplitudes along the $x$ direction (${t}_{\perp }$), along the $y$ direction (${t}_{\parallel }$), the next-nearest hopping along the diagonal (${t}_{D}$), as well as the onsite potentials ${\epsilon }_{i}$. Clearly the dispersion along the x direction vanishes as we approach smaller angles. The results of the fit are summarized in Table 2.

Fitting the bands for three different twist angles $\phi =10.9{9}^{\circ }$, $\phi =8.2{6}^{\circ }$, and $\phi =6.6{1}^{\circ }$ yields the values reported in Table 2. Clearly, as one approaches smaller twist angles the one-dimensional character of the system emerges and the residual chain-chain coupling along the x direction ${t}_{\perp }$ and ${t}_{D}$ becomes negligible. This is further illustrated in Fig. 5 where we show the ab initio characterization of the dispersion for the same angles, as well as the corresponding fits. The bands show more appreciable residual dispersion along the $X-S$ direction at larger angle, signaling the crossover from 1D to 2D as the angle is increased. Therefore the effective dimensionality of the system can be tuned by the twist angle and twisted bilayer GeSe provides a tunable platform to study the 2D to 1D crossover.

**Table 2:** Fitted values for the tight binding model of Eq. (3) for three different angles as shown in Fig. 5.

$\phi$	${t}_{\parallel }$	${t}_{\perp }$	${t}_{D}$	${\epsilon }_{0,0}$	${\epsilon }_{0,1}$	${\epsilon }_{1,0}$	${\epsilon }_{1,1}$
10.99	7.024	0.992	0.039	408.359	406.214	407.522	404.923
8.26	2.403	0.094	0.037	369.076	370.521	370.474	371.556
6.61	1.349	0.017	0.036	353.57	355.048	355.249	353.135

Clearly the system becomes more one-dimensional as the angle becomes smaller.

All values are given in meV.

For the smallest twist angle of $\phi =6.6{1}^{\circ }$, which we concentrate on in the main text when discussing correlation effects, the dispersion along $x$ is negligible and we can set ${t}_{\perp }\approx 0$, ${t}_{D}\approx 0$, as well as label $t={t}_{\parallel }$. Subtracting of the trivial mean potential shift of ${\epsilon }_{{\rm{s}}hift}=({\epsilon }_{x,0}+{\epsilon }_{x,1})/2$ and defining ${\epsilon }_{0}=({\epsilon }_{x,0}-{\epsilon }_{x,1})/2$ we recover Eq. (1) of the main text (as well as reinstating the spin degree of freedom).

### Treating electron correlations

We treat correlations in a numerically exact tensor network based approach formulated in matrix product states^16^. We exploit the two-site translation invariance of the infinite system and set up the tensor network algorithm directly for the infinite dimensional limit. To treat finite temperature we use the purification scheme described in part 7 of ref. ^16^ and rewrite the unity operator, corresponding to an infinite temperature density matrix $\rho \sim 1$ in terms of a wavefunction in combined physical and auxiliary Hilbert space. Subsequently we “cool” the density matrix to temperature $T=1/\beta$, where $\rho \sim {e}^{-\beta H}$, by applying an imaginary time evolution algorithm. We converge the bond dimension such that numerically exact results are obtained and perform a fourth order Trotter-Suzuki decomposition with small enough steps in imaginary time $\Delta \beta =0.01$, such that the decomposition does not yield an appreciable approximation. A fourth order decomposition is chosen for numerical convenience allowing for larger time steps then a second order scheme reducing the overall numerical resources needed. In the Supplementary Note 2 the convergence of all numerical parameters is benchmarked explicitly in the non-interacting limit.

### Calculating the density of states

To calculate the density of states we use a simulation in real time (and at finite temperature) to obtain the

4 $$G(t)=\left\langle {c}_{i,\uparrow }{c}_{i,\uparrow }^{\dagger }(t)\right\rangle.$$

**Fig. 6:**
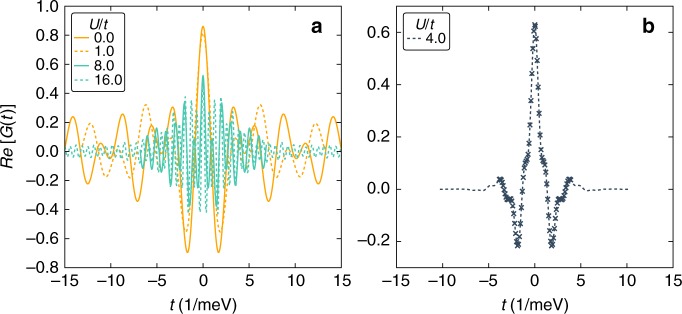
Real time simulation of $G(t)$ (see Eq. (4)) of the data shown in Fig. 3. Panel **a** showns $U/t=0$, 1, 8 and 16, while panel **b** displays $U/t=4$. In the case of $U/t=4$ we extend the reached time scales by using linear prediction. Symbols are calculated data points, the line is the data obtained using linear prediction.

For this we use the ideas put forward in ref. ^56^. This is essential to reach long enough times, such that a meaningful Fourier transform can be taken with a Hanning type window function, compare Fig. 6a. The maximum time reached by the simulation thus limits the frequency resolution and introduces natural broadening in the Fourier transform. This procedure is employed for the Data shown in Fig. 3a, c where the $U/t$ is either large or small both cases in which the entanglement growth is quite moderate. For the data shown Fig. 3b which is $U/t=4$ the entanglement growth is much more severe and even after employing the ideas of ref. ^56^, the time scales are limited. To this end we utilize a linear prediction algorithm to extend the time scales, see Fig. 6b.

## Supplementary information

Supplementary Information

## Data Availability

All data generated and analyzed during this study are available from the corresponding author upon reasonable request.

## Code availability

All the custom codes used in this study are available from the corresponding author upon reasonable request.
