# Supplementary material for: One-dimensional flat bands in twisted bilayer germanium selenide
Source: Nat Commun. 2020 Feb 28;11:1124. doi: 10.1038/s41467-020-14947-0 (PMC7048812; doi:10.1038/s41467-020-14947-0)
Supplement: Supplementary file 1 — Supplementary Information [file 41467_2020_14947_MOESM1_ESM.pdf]

Supplementary Information – One-dimensional flat bands in twisted bilayer germanium selenide

D. M. Kennes,[1,2] L. Xian,[2] M. Claassen,[3] and A. Rubio[2,3,4]

[1] *Institut für Theorie der Statistischen Physik, RWTH Aachen University and JARA-Fundamentals of Future Information Technology, 52056 Aachen, Germany*

[2] *Max Planck Institute for the Structure and Dynamics of Matter, Center for Free Electron Laser Science, 22761 Hamburg, Germany*

[3] *Center for Computational Quantum Physics, Simons Foundation Flatiron Institute, New York, NY 10010 USA*

[4] *Nano-Bio Spectroscopy Group, Departamento de Fisica de Materiales, Universidad del País Vasco, UPV/EHU- 20018 San Sebastián, Spain*

(Dated: January 31, 2020)

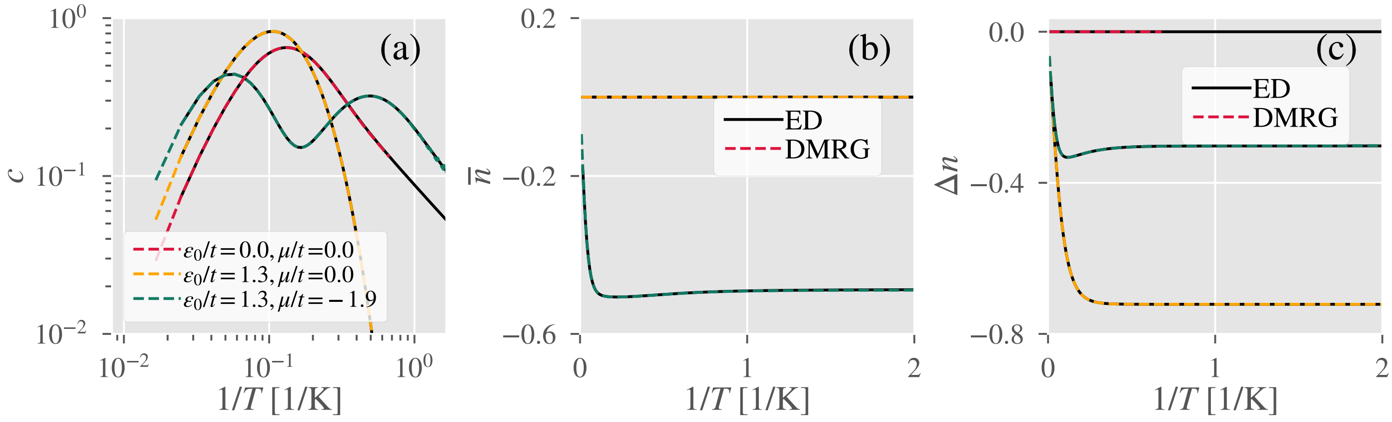

Supplementary Fig. 1. Benchmarking the DMRG against exact solution (ED) at $U = 0$ (a) specific heat (b) average occupancy (c) difference in occupancy between even and odd lattice sites. We show that numerical convergence to the obtain results which are numerically exact can be achieved.

SUPPLEMENTARY NOTE 1: CHARACTERIZATION OF PHASES

In the main text we use small seed fields to efficiently characterize susceptibilities towards the different ordering tendencies of the Ionic Hubbard model. Here we present the details of the calculation for completeness. We define the susceptibility

$$\chi^X = O/s \tag{1}$$

as the ratio between an appropriately chosen observable (measuring the symmetry breaking accompanied by the phase) and the strength s of a symmetry breaking seed field ΔH^X added to the Hamiltonian. For the magnetization and charge susceptibilities $X = M$ and $X = C$ we chose O as the magnetization $M = \sum_{i,\sigma}(-1)^\sigma n_{i,\sigma}/N$ or charge $C = \sum_{i,\sigma} n_{i,\sigma}/N$. The seeds added to the Hamiltonian are $\Delta H^M = s\sum_{i,\sigma}(-1)^\sigma n_{i,\sigma}$ and $\Delta H^C = s\sum_{i,\sigma} n_{i,\sigma}$. For the susceptibility to BOW ordering $X = BOW$ we chose O as the dimerization in the hopping $B = \sum_{i,\sigma}(-1)^i c^\dagger_{i,\sigma}c_{i+1,\sigma}/N$ and the seed as $\Delta H^{BOW} = s\sum_{i,\sigma}(-1)^i c^\dagger_{i,\sigma}c_{i+1,\sigma}$.

SUPPLEMENTARY NOTE 2: BENCHMARKING THE DMRG WITH EXACT SOLUTIONS

In supplementary figure 1 we benchmark our thermodynamic limit finite temperature DMRG results against exact results obtained in the non-interacting limit $U = 0$ of equation (1) in the main text. We calculate the specific heat (as in the main text), the average occupancy $\bar{n} = \lim_{N\to\infty}\sum_i n_i/N$ as well as the difference in occupancy between even and odd lattice sites $\Delta n = \lim_{N\to\infty}\sum_i(-1)^i n_i/N$. We show that we can converge the numerical parameters to obtain results which are numerically exact.
